# Identification of Small Molecule Inhibitors of *Clostridium perfringens* ε-Toxin Cytotoxicity Using a Cell-Based High-Throughput Screen 

**DOI:** 10.3390/toxins2071825

**Published:** 2010-07-09

**Authors:** Michelle Lewis, Charles David Weaver, Mark S. McClain

**Affiliations:** 1 Vanderbilt Institute of Chemical Biology, Vanderbilt University, Nashville, TN 37235, USA; Email: michelle.lewis@vanderbilt.edu; 2 Department of Pharmacology, Vanderbilt University, Nashville, TN 37235, USA; Email: david.weaver@vanderbilt.edu; 3 Department of Medicine, Vanderbilt University, Nashville, TN 37235, USA

**Keywords:** bacterial toxins, *Clostridium perfringens*, cell membrane permeability, small molecule libraries, structure-activity relationship, drug evaluation, preclinical

## Abstract

The *Clostridium perfringens* epsilon toxin, a select agent, is responsible for a severe, often fatal enterotoxemia characterized by edema in the heart, lungs, kidney, and brain. The toxin is believed to be an oligomeric pore-forming toxin. Currently, there is no effective therapy for countering the cytotoxic activity of the toxin in exposed individuals. Using a robust cell-based high-throughput screening (HTS) assay, we screened a 151,616-compound library for the ability to inhibit ε-toxin-induced cytotoxicity. Survival of MDCK cells exposed to the toxin was assessed by addition of resazurin to detect metabolic activity in surviving cells. The hit rate for this screen was 0.6%. Following a secondary screen of each hit in triplicate and assays to eliminate false positives, we focused on three structurally-distinct compounds: an *N*-cycloalkylbenzamide, a furo[2,3-*b*]quinoline, and a 6*H*-anthra[1,9-*cd*]isoxazol. None of the three compounds appeared to inhibit toxin binding to cells or the ability of the toxin to form oligomeric complexes. Additional assays demonstrated that two of the inhibitory compounds inhibited ε-toxin-induced permeabilization of MDCK cells to propidium iodide. Furthermore, the two compounds exhibited inhibitory effects on cells pre-treated with toxin. Structural analogs of one of the inhibitors identified through the high-throughput screen were analyzed and provided initial structure-activity data. These compounds should serve as the basis for further structure-activity refinement that may lead to the development of effective anti-ε-toxin therapeutics.

## 1. Introduction

The *C. perfringens*ε-toxin is a potent bacterial protein toxin, with an LD_50_ in mice on the order of 100 ng per kg [1,2], and is categorized by the US Department of Health and Human Services as a select agent. The ε-toxin is responsible for a severe enterotoxemia primarily in livestock animals but may also be toxic to humans [3,4,5,6,7]. As is true of many select agents and toxins, human exposure to ε-toxin appears to be rare. Pathologic changes are observed primarily in the brains and kidneys of animals intoxicated by ε-toxin [8,9,10], but edema of the heart and lungs is also common. 

Following interaction with sensitive cells, ε-toxin assembles into heptameric oligomers [11,12]. Evidence from multiple studies suggests that these ε-toxin oligomers form pores in the plasma membrane of cells. Addition of purified toxin to cells is followed rapidly by efflux of intracellular K^+^ and increases in intracellular Cl^−^ and Na^+^ [13,14]. MDCK cells treated with ε-toxin become permeable to fluorescent dyes including propidium iodide and 7-aminoactinomycin D (7-AAD), with a strong correlation between the kinetics of dye entry and loss of cell viability [14,15,16]. Experiments using artificial lipid bilayers confirmed the channel-forming properties of the toxin [14], and there is no evidence that ε-toxin enters cells [13,17,18]. Thus, it is widely believed that ε-toxin acts by forming ion-permeable pores in the plasma membrane of sensitive cells. 

Both a vaccine and anti-toxin immune sera are approved for veterinary use in the protection of economically-important livestock, where (for example) ε-toxin may account for over 20% of deaths among feedlot sheep [19]. However, neither the vaccine nor the anti-toxin immune sera is effective at treating animals intoxicated by ε-toxin and no therapy for ε-toxin-related illness is approved for human use. The veterinary vaccine is effective at providing immunity to the toxin, though a series of booster immunizations may be required to confer long-term immunity [20]. Anti-toxin immune sera may also be used to protect immunologically naïve animals. However, in the event that an animal exhibits symptoms of intoxication by ε-toxin, it is typically too late for the current anti-toxin to be effective and use of the anti-toxin is typically limited to prophylactic treatment of unvaccinated animals within a herd [21]. Thus, both of the existing approaches to combat ε-toxin that are approved for veterinary use are of limited value in treating individuals exposed to ε-toxin. Alternative countermeasures are needed that inhibit the activity of the toxin. Unlike existing vaccine and antitoxin therapies, a small molecule inhibitor may be effective in inhibiting ε-toxin after the toxin has bound to cells by blocking the pore-forming properties of the toxin or by blocking a host response that leads to cell death. Such an inhibitor could be an effective treatment for individuals exposed to the toxin.

In this study, we carried out a high-throughput cell-based screen to identify small molecule inhibitors of the *C. perfringens*ε-toxin. The inhibitory activities of these compounds and structural analogs were verified using a variety of different measures of cytotoxic activity.

## 2. Materials and Methods

### 2.1. Cell culture

MDCK cells (ATCC) were routinely cultured in MEM supplemented with glutamax (Invitrogen) and 10% fetal clone III (HyClone) and incubated at 37 °C in 5% CO_2_. Cells were subcultured by diluting two-fold into fresh medium 16 to 24 hours prior to cytotoxicity screens.

### 2.2. Recombinant ε-toxin expression and purification

The gene encoding ε-prototoxin, *etxB*, was fused to a carboxy-terminal 6-His tag and cloned into pET22b [22]. The ε-prototoxin expressing plasmid was transformed into NovaBlue (DE3) (Novagen), along with the plasmid pLysE (encoding bacteriophage T7 lysozyme) and transformants were grown in broth supplemented with antibiotics to an optical density at 600 nm of 0.7. Isopropyl β-D-thiogalactopyranoside then was added to a final concentration of 1 mM to induce expression of the cloned gene, and the cultures were grown for another 3 h. The cells were collected and resuspended in 1/20th culture volume of B-PER Bacterial Protein Extraction Reagent (Pierce) supplemented with Complete Mini protease inhibitor cocktail (EDTA-free, Roche), and mixed for 10 minutes at room temperature. The cell debris was pelleted, and the supernatant was recovered. The B-PER extracted material was diluted four-fold with water, and applied to a Q-Sepharose column. The ε-prototoxin-containing flow-through material was collected and applied to a Ni-NTA affinity column (Qiagen). The Ni-NTA column was washed with a buffer comprised of 20 mM sodium phosphate, 300 mM sodium chloride, and 20 mM imidazole (pH 8.0), and the ε-prototoxin was eluted in a buffer comprised of 20 mM sodium phosphate, 300 mM sodium chloride, and 250 mM imidazole (pH 8.0). The identification of the ε-prototoxin protein in the purified sample was confirmed by immunoblotting with an ε-toxin-specific monoclonal antibody [23]. A derivative plasmid that expressed a GFP-ε-toxin fusion protein was also used [21]. Protein concentrations were determined using micro-BCA (Pierce).

### 2.3. Compound library

A compound library consisting of 151,616 compounds represents a diverse subset of the >1 million member inventories of two companies, ChemDiv and ChemBridge. Each chemical is dissolved at a concentration of 10 mM in dimethylsulfoxide. Confirmed hits and their structural analogs were acquired from commercial sources (ChemDiv, ChemBridge, and Specs) as dry powders and dissolved in DMSO.

### 2.4. High-throughput screen for ε-toxin inhibitors

Assays were performed using black, clear bottom 384-well plates (Greiner Bio-One). Compounds (10 nL of 10 mM stock solutions in DMSO) were dispensed using an Echo 550 non-contact liquid handler (Labcyte). Medium (10 μL phenol red-free Leibovitz L-15 supplemented with glutamax) was added to each well using a Multidrop liquid handler (Thermo Electron). The assay plates then were transported to a biological containment lab for further processing.

Within the biological containment lab, 10 μL of 5 × 10^5^ MDCK cells per mL (in phenol red-free Leibovitz L-15 medium supplemented with glutamax, 10% fetal clone III, and antibiotic-antimycotic solution (Invitrogen)) were added to the wells of the assay plates using a BioTek μFill dispenser. Purified recombinant ε-toxin (in Leibovitz L-15, 10 μL per well, at a final concentration of 10 nM) then was added to each well using a BioTek μFill dispenser. Plates were incubated at 37 °C for 16 hours. Cytotoxicity was assessed by detecting metabolically active cells. CellTiter Blue reagent (10 μL per well, Promega) was added to the assay plates, and the plates were incubated at 37 °C for an additional 4 hours. Fluorescence at 590 nm was measured following excitation at 560 nm using a BioTek FLx800 plate reader. Control plates lacking library compounds were included at the beginning and end of each set of assay plates. One half of each control plate received medium without toxin (*i.e*., cells only) whereas the remaining halves of each control plate received medium containing toxin. If Z’ for either of the two control plates was less than 0.5, the data set was discarded and the compounds were re-evaluated [24].

### 2.5. Secondary screens

Compounds that appeared to inhibit ε-toxin-induced cytotoxicity in the primary HTS were re-tested in triplicate as described above, except that the ε-toxin concentration was increased to 20 nM. Compounds exhibiting the greatest apparent inhibitory activity upon re-testing in triplicate were examined for non-specific assay interference by adding the compounds to cells in the absence of ε-toxin. Compounds that led to a significant increase in fluorescence at 590 nm were excluded from further study.

### 2.6. Immunoblotting

Protein samples in SDS sample buffer were heated in a boiling water bath for 5 min before analysis by SDS-PAGE and transferred to nitrocellulose membranes. Membranes were developed using anti-GFP (Santa Cruz Biotechnology, SC-9996) or anti-beta-actin (Abcam, ab8227) antibodies followed by horseradish peroxidase-conjugated secondary antibodies. SuperSignal West Femto or SuperSignal West Pico substrates (Pierce) were used for enhanced chemiluminescent detection.

### 2.7. Propidium iodide influx

MDCK cells (10^4^ cells per well) were plated in black, clear-bottomed 96-well plates. Propidium iodide (5 μg per mL, final concentration), ε-toxin, and inhibitory compounds were added (as appropriate) to the cells and fluorescence at 645 nm was measured following excitation at 485 nm using a BioTek FLx800 plate reader at 37 °C.

### 2.8. Select agent

Plasmid DNA capable of expressing the ε-prototoxin (or ε-toxin) is considered a select agent by the U.S. Department of Health and Human Services.

## 3. Results and Discussion

### 3.1. Assay validation

**Figure 1:**
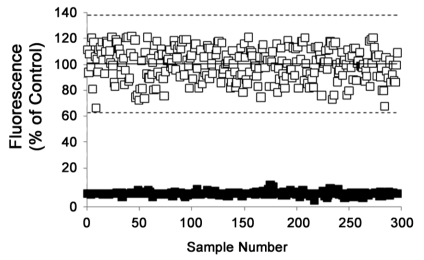
Assay validation.MDCK cells were treated with purified ε-toxin (■) or with medium alone (□) and incubated for 16 hours at 37 °C. CellTiter Blue then was added, and the cells incubated for an additional 4 hours. The fluorescent signal then was measured using a FLx800 plate reader. Results from two independent assays performed on different days were normalized to 100% (mean of cells treated with medium alone) and 0% (mean of cells treated with ε-toxin). Individual wells were arbitrarily assigned a sample number in order to plot the results. The means (solid line) and mean ± three-times the standard deviation (dashed line) are shown. Z’ for the combined data was 0.592.

We modified an MTT-based assay used to determine ε-toxin-induced cytotoxicity for use in a high-throughput screen (HTS) [22,25]. We scaled the assay to use 384-well plates instead of 96-well plates, and replaced the colorimetric indicator MTT with the fluorescent metabolic indicator resazurin (CellTiter Blue, Promega) [22]. Metabolically-active cells reduce resazurin to the fluorescent compound resorufin. Preliminary experiments were used to maximize the dynamic range (difference in fluorescence between toxin-treated and untreated MDCK cells) of the assay by determining the optimal number of cells per well and dose of ε-toxin needed to kill greater than 90% of the cells. Well-to-well variability of the assay was minimized through the use of automated fluid dispensers. To assess the suitability of the assay for use in a HTS, MDCK cells were dispensed into 384-well plates. Medium alone or medium containing purified ε-toxin then was added to the cells and cytotoxicity was assessed as described. A statistic, the Z' factor, accounts for both the dynamic range and the amount of variability in an assay, and is frequently used to assess assay performance [24]. Assays with Z' factors between 0.5 and 1 are typically considered to be excellent assays and to be suitable for HTS. Using control plates containing MDCK cells in medium alone and MDCK cells treated with ε-toxin, the modified assay routinely exhibits Z' values greater than 0.5 (Figure 1). Additional experiments demonstrated that this assay is insensitive to DMSO (the solvent in which the small drug-like molecules have been dissolved) concentrations up to 1% (data not shown). 

### 3.2. High-throughput screen

The Vanderbilt Institute of Chemical Biology HTS facility has chemical libraries from two sources, ChemDiv and ChemBridge. The 151,616 chemicals screened in this assay are representative of the chemical diversity of the larger ChemDiv and ChemBridge compound collections. To screen the chemical library, MDCK cells and ε-toxin were added to 384-well plates containing library compounds (1 compound per well) and incubated for 16 hours at 37 °C (Materials and Methods). CellTiter Blue reagent then was added to assess cell viability. Fractions of the library were screened on 11 different days over a 10 week period. Each assay included control plates of cells with and without ε-toxin (no compounds) positioned at the beginning and end of each set of compound-containing plates. The mean Z' calculated from the control plates over the course of the entire screen was 0.653. 

**Figure 2:**
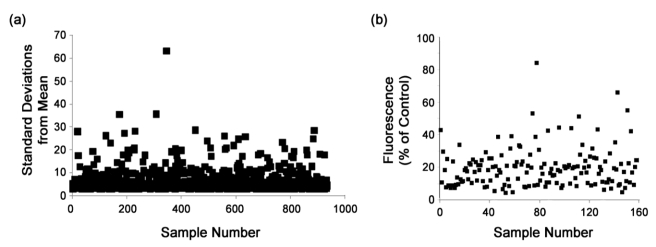
Compound Screening. (**a**)Primary HTS. Results represent the number of standard deviations by which the B-score for each compound differs from the mean B-score; results are limited to 915 out of 151616 compounds tested for which the B-score was greater than three standard deviations from the mean B-score. Individual compounds were arbitrarily assigned a sample number in order to plot the results; (**b**) Secondary screen. Results were normalized to the fluorescent signal from MDCK cells that received compounds but no ε-toxin (100%) and cells treated with toxin in the absence of compounds (0%). Data represent the mean and standard deviation of at least quadruplicate samples and are limited to 158 out of 915 compounds tested for which the z-score was greater than three. Individual compounds were arbitrarily assigned a sample number in order to plot the results.

To identify active compounds (e.g., compounds that inhibit ε-toxin cytotoxicity), raw fluorescent data was converted to B-scores using HTS-Corrector [26]. The B-score is an analog of the common practice of determining the number of standard deviations by which a signal differs from the mean (*i.e*., normal deviate or z-score), but makes minimal assumptions concerning the distribution of the data, corrects for row-to-row and column-to-column systematic variability by fitting a two-way median polish [27], and is resistant to statistical outliers [28,29]. We defined a hit as a compound that yielded a B-score that was more than 3 standard deviations greater than the mean B-score [28,30]. Using this approach, we identified 915 hits from screening the 151,616-compound library for a hit rate of 0.6% (Figure 2a).

### 3.3. Secondary screens

Hits identified in the initial screen were retested in triplicate as a secondary screen (each compound was tested once in the primary HTS). The mean fluorescent signal for each compound was compared to the mean fluorescent signal from control wells containing MDCK cells treated with ε-toxin, but with no compound added. Confirmed hits were defined as compounds for which the mean fluorescent signal was greater than the mean signal of the control wells by greater than three standard deviations (*i.e*., z-score > 3) [28]. Results indicated that 17% (158 of 915) of the retested compounds were inhibitory. The low confirmation rate was likely due, at least in part, to the higher dose of toxin used in re-testing (Materials and Methods). The fluorescent signal from cells treated with the confirmed hits ranged from 4 to 84% of control (Figure 2b). Although the level of inhibition exhibited by many of these compounds may not be biologically relevant, the purpose of a high-throughput screen is not to provide drug candidates *per se*, but rather to provide chemical information necessary to infer structure-activity relationships that then will be used to develop drug candidates. 

To further study confirmed hits, the compounds were ranked by percent inhibition. Eighteen of the 20 most active compounds were commercially available and were acquired as powders. The fluorescent signal from cells treated with these compounds ranged between 32 and 84% of control in the secondary screen. The powdered compounds were dissolved in DMSO. Because the primary and secondary HTS assays lacked a counterscreen to identify compounds affecting the CellTiter Blue assay, the acquired compounds were incubated with cells in the absence of toxin and the cells were treated with CellTiter Blue reagent. Compounds that led to an elevated fluorescent signal were considered to be false positives. The false positives included members of two distinct structural families, representing 11 of the 20 most active compounds (including the two most active compounds). Members of these two structural families were excluded from further analysis; 7 of the 18 compounds we acquired did not interfere with the CellTiter Blue assay. 

### 3.4. Analysis of confirmed hits

**Figure 3:**
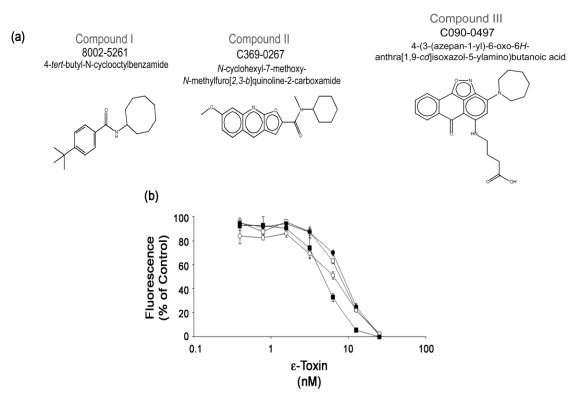
Confirmed Hits. (**a**) The ChemDiv compound numbers, names, and structures of three confirmed hits are shown; (**b**) Serial dilutions of ε-toxin (25 to 0.39 nM) were added to MDCK cell monolayers in a 384-well plate, in the presence of 50 μM inhibitory compounds in 0.4% DMSO (□: compound I; ●: compound II; ○: compound III) or 0.4% DMSO as a control (■). After 16 hours incubation, CellTiter Blue (Promega) was added. Results were normalized to the fluorescent signal from MDCK cells that received compounds but no ε-toxin (100%) and cells treated with compounds and 0.1% Triton (0%). Data represent the mean and standard deviation of at least quadruplicate samples. The CT_50_ values (4.7 nM, DMSO control; 7.3 nM, compound I; 6.5 nM, compound II; and 6.7 nM, compound III) were calculated by non-linear regression analysis. CT_50_ values for cells treated with compounds were significantly increased compared to the CT_50_ value of cells treated with the DMSO control (ANOVA followed by Dunnett's post hoc test, P < 0.05).

We sought to determine the compound concentrations that produced minimal and maximal inhibition of ε-toxin-induced cytotoxicity (*i.e.*, I_min_ and I_max_, respectively). Based on I_min_ and I_max_ we could estimate the compound concentration that inhibited ε-toxin-induced cytotoxicity by 50% (*i.e.*, IC_50_). However, due to limited solubility of the seven compounds in the tissue culture medium, we were unable to determine I_max_. Maximum effects frequently cannot be obtained due to poor compound solubility [31,32]. As an alternative to compound IC_50_ determinations, we determined the extent to which a fixed dose of the compounds increased the concentration of toxin needed to kill 50% (CT_50_) of the MDCK cell monolayer. Based on these results, we selected three compounds (yielding the greatest increase in CT_50_) for further study (Figure 3). The selected compounds represent three distinct structural families, including an *N*-cylcoalkylbenzamide, a furo[2,3-*b*]quinoline, and a 6*H*-anthra[1,9-*cd*]isoxazol. Each of the compounds selected for further study yielded a significant increase in the toxin CT_50_. The fluorescent signal from cells treated with these three compounds was 51, 34, and 38% of control, respectively, in the secondary screen (Figure 2b).

**Figure 4:**
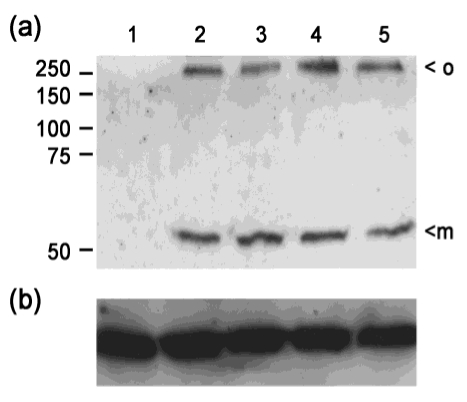
Toxin oligomerization. MDCK cells treated at 37 °C for 30 min with medium alone (lane 1), or medium containing GFP-tagged ε-toxin (17 nM final concentration) in the presence of DMSO (lane 2), 50 μM compound I (lane 3), compound II (lane 4) or compound III (lane 5) were washed to remove unbound toxin and lysates were prepared. The samples were analyzed by SDS-PAGE and immunoblotting with anti-GFP (**a**) or anti-actin (**b**) antibodies as a loading control. Molecular weight markers, the GFP-tagged ε-toxin monomer (m), and heat- and SDS-resistant oligomers of the GFP-tagged ε-toxin (o) are indicated.

We next sought to determine if the inhibitory compounds interfered with the ability of ε-toxin to bind to cells or to form oligomeric complexes on cells. Toxin binding and oligomerization can be detected by immunoblotting, though we do not have an anti-ε-toxin antibody that readily detects the toxin in association with MDCK cells. To overcome this obstacle, we expressed, purified, and trypsin-treated a GFP-tagged form of the wild-type ε-toxin [8,9,33]. This toxin retains cytotoxic activity, the GFP tag is not lost following trypsin treatment, and the GFP-ε-toxin fusion protein can be detected on cells using an anti-GFP antibody. The GFP-tagged ε-toxin was incubated with MDCK cells at 4 °C in the presence or absence of the inhibitory compounds for 1 hour. The cells then were washed to remove unbound toxin and cell lysates were prepared. An immunoblot with anti-GFP antibody revealed no detectable difference in the amount of ε-toxin bound to cells (data not shown). To determine if the inhibitory compounds disrupted the ability of ε-toxin to form oligomeric complexes, the GFP-tagged ε-toxin was incubated with MDCK cells at 37 °C in the presence or absence of the inhibitory compounds for 30 minutes. Under these conditions, ε-toxin forms heat- and SDS-resistant oligomeric complexes believed to represent the membrane-inserted pore structure. The cells then were washed to remove unbound toxin and cell lysates were prepared. An immunoblot with anti-GFP antibody revealed no detectable difference in the ability of the GFP-tagged ε-toxin to assemble into oligomeric complexes (Figure 4). These results suggest that the inhibitory compounds interfere with either the channel formed by the toxin or with unidentified host factors that may contribute to ε-toxin-induced cytotoxicity.

**Figure 5:**
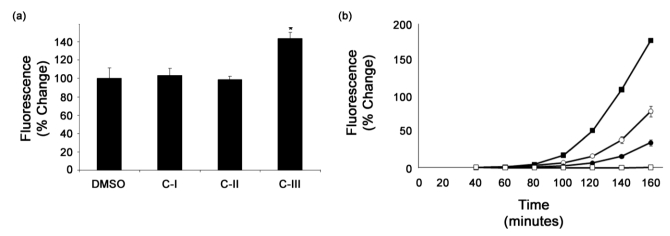
Inhibition of propidium iodide influx. (**a**) MDCK cells in 96-well plates were overlayed with Leibovitz L-15 medium containing 5 μg per mL propidium iodide in the presence or absence of 0.1% Triton X-100 at 37 °C. Medium was supplemented with either DMSO (control), or compounds I, II, or III. The final concentration of compound I, II, and III was 50 μM, and the final DMSO concentration in all wells was 0.4%. Cells were incubated at 37 °C for 30 minutes. The percent change in fluorescence was calculated by subtracting the fluorescent signal in the absence of detergent from the fluorescent signal of detergent-treated samples and normalizing this net fluorescence value to the net fluorescence of cells treated with DMSO. Results indicate the mean and standard deviation of at least quadruplicate samples. The asterisk indicates results significantly different from the DMSO control (ANOVA followed by Dunnett's post hoc test, P < 0.05); (**b**) MDCK cells in 96-well plates were incubated in Leibovitz L-15 medium containing 5 μg per mL propidium iodide at 37 °C. At time zero, triplicate wells received ε-toxin and DMSO (■), DMSO only (□), ε-toxin and compound I (●), or ε-toxin and compound II (○). The final toxin concentration was 25 nM. The final concentration of compound I and compound II was 50 μM, and the final DMSO concentration in all wells was 0.4%. Fluorescence was measured at 20 minute intervals. Results represent the mean % change in fluorescencefrom the 40 minute reading (to confirm the signal had stabilized) based on triplicate samples.

As an independent means to assess the inhibitory activity of the selected compounds, we sought to determine whether the compounds could interfere with ε-toxin-induced permeabilization of MDCK cells. Previous studies have demonstrated that the plasma membrane of cells exposed to ε-toxin becomes permeable to molecules up to 1 kDa in size [14,16]. This membrane permeability can be detected by incubating cells with propidium iodide (which is generally excluded from viable cells, and which exhibits a 20- to 30-fold increase in fluorescence upon binding nucleic acids) [14,15]. We first assessed whether the selected compounds exhibited an adverse effect on propidium iodide fluorescence. Cells were treated with propidium iodide in the presence of DMSO (control) or each of the selected compounds in the presence or absence of detergent to permeabilize the plasma membrane. Results indicated that compounds I and II did not interfere with the measurement of propidium iodide fluorescence; compound III interfered with the fluorescence of propidium iodide and was not evaluated for the ability to inhibit ε-toxin-induced dye uptake by MDCK cells (Figure 5a). We next determined whether compounds I and II inhibited the uptake of propidium iodide by MDCK cells in response to ε-toxin. Cells were treated with medium alone, or with toxin-containing medium supplemented with DMSO (control), compound I, or compound II. Results indicated that compounds I and II inhibited the uptake of propidium iodide by the MDCK cells (Figure 5b). 

**Figure 6:**
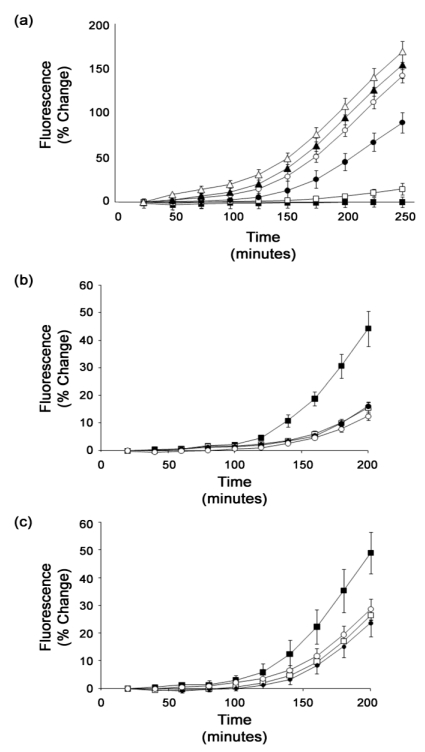
Inhibition of propidium iodide influx in cells pre-treated with ε-toxin. (**a**) MDCK cells in Leibovitz L-15 medium were treated with 25 nM ε-toxin at 37 °C for 0 (■), 5 (□), 15 (●), 25 (○), 30 (▲), or 35 (∆) minutes before unbound toxin was washed away and fresh medium containing 5 μg per mL propidium iodide was added (time 0). Fluorescence was measured at 12 minute intervals. Results represent the mean % change in fluorescence from the 24 minute reading (to confirm the signal had stabilized) based on triplicate samples. (**b**) and (**c**) MDCK cells in Leibovitz L-15 medium were treated with 25 nM ε-toxin at 37 °C for 10 minutes before unbound toxin was washed away and fresh medium containing 5 μg per mL propidium iodide was added (time 0). Medium containing propidium iodide and DMSO was added at time 0 (■). Medium containing propidium iodide and compound I (**b**) or compound II (**c**)was added at 0 (□), 5 (●), or 10 (○) minutes after toxin removal. The final toxin concentration was 25 nM. The final concentrations of compound I and compound II was 50 μM, and the final DMSO concentration in all wells was 0.4%. Fluorescence was measured at 20 minute intervals. Results represent the mean % change in fluorescence from the 20 minute reading based on triplicate samples.

To further explore the inhibitory activity of the compounds, we next examined the activity of the inhibitors towards cells pre-treated with ε-toxin. MDCK cells were treated with ε-toxin for various times before the cells were washed to remove unbound toxin, and propidium iodide was added. Results indicated that cells became permeable to propidium iodide after being exposed to the toxin for as little as 5 minutes (Figure 6a). To determine whether compound I or II might be effective on cells previously exposed to ε-toxin, MDCK cells were treated with ε-toxin for 10 minutes before being washed to remove unbound toxin and fresh medium containing propidium iodide was added. The inhibitory compounds then were added at various times after toxin removal. Results indicated that compounds I and II were active when added to cells at least 10 minutes after toxin had been removed from the cells (Figure 6b and c). 

### 3.5. Structure-activity relationships

We next sought to explore structure-activity relationships of these compounds by acquiring and testing a variety of structural analogs. Due to the limited availability of analogs to compounds II and III, we limited this analysis to analogs of compound I. Forty-three structural analogs of compound I were tested at 25 μM final concentration (using the CellTiter Blue assay) and z-scores were determined by comparison to control cells treated with ε-toxin alone (Supplemental Table 1). Seven of the analogs yielded z-scores greater than 3 and were considered to inhibit ε-toxin-induced cytotoxicity [28]. We then determined the extent to which a fixed dose of the inhibitory compounds increased the concentration of toxin needed to kill 50% (CT_50_) of an MDCK cell monolayer. Results revealed a variety of inhibitory activities depending on the various substitutions the core structure (Table 1). 

**Table 1:** Inhibitory activity of compound I and structural analogs.

**Compound**	**CT_50_ (25 μM) ^a^**	***P* < 0.05 (25 μM) ^b^**	**CT_50_ (50 μM) ^a^**	***P* < 0.05 (50 μM) ^b^**
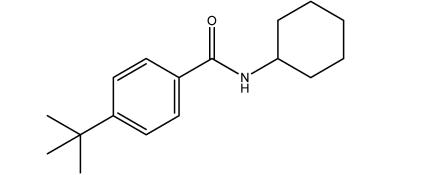	8.1	Yes	10.1	Yes
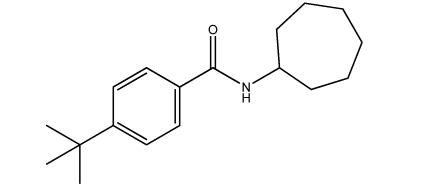	7.2	Yes	9.1	Yes
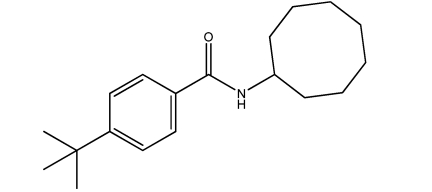	7.4	Yes	8.5	Yes
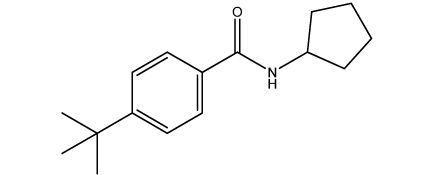	6.1	Yes	7.2	Yes
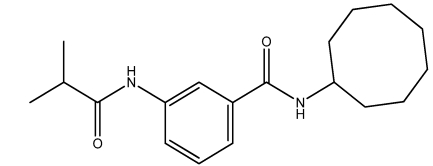	5.9	Yes	6.7	Yes
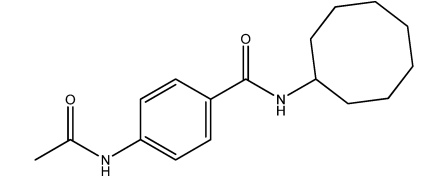	5.8	Yes	6.6	Yes
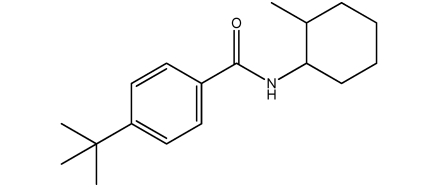	4.7	No	5.7	No
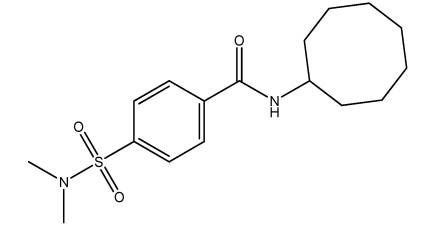	5.1	No	5.7	No
No compound	4.4	-	4.8	-

^a^ The inhibitory compounds were added to the medium overlying MDCK cells at either 25 or 50 μM final concentration. Serial dilutions of ε-toxin were added and the plates incubated for 16 hours at 37 °C. Cell viability was determined with CellTiter Blue as described (Materials and Methods). The CT_50_ of each compound was determined by non-linear regression analysis of quadruplicate samples at each dose of toxin.

^b^ The CT_50_ determined in the presence of each inhibitory compound was compared to the CT_50_ of cells treated with no compound (ε-toxin and DMSO vehicle control) by ANOVA followed by Dunnett’s post-hoc test.

## 4. Conclusions

Conventional therapeutics (vaccines or anti-toxin immune serum) are of limited use in the treatment of many toxin-mediated illnesses once an individual exhibits symptoms of toxin exposure, likely because the events leading to cytotoxicity have already begun [34]. New therapeutics are needed that can inhibit the activities of toxins that have already interacted with host cell targets. Several studies report identification of toxin inhibitors by screening small molecule libraries. Using direct screens for inhibitors of toxin activity, small molecule inhibitors have been identified that block the interaction between *Bacillus anthracis* edema factor and the host activator protein calmodulin [35], that inhibit the adenylate cyclase activities of edema factor and the CyaA toxin of *Bordetella pertussis* [36], that inhibit the protease activities of *B. anthracis* lethal factor [37] and *Clostridium botulinum* neurotoxin A [38], that inhibit the internalization of anthrax toxin [39], that inhibit intracellular transport of a variety of toxins acting intracellularly [40], and that inhibit the activities of ricin and Shiga toxins [41]. Indirectly, small molecule inhibitors of the cystic fibrosis transmembrane conductance regulator are able to reduce fluid secretion resulting from exposure to cholera toxin [42], and inhibitors of the cell surface protease furin have been shown to inhibit the activation of protective antigen [43]. To our knowledge, this is the first report utilizing a high-throughput screen to identify small molecule inhibitors of a bacterial pore-forming toxin.

The compounds we focused on in the present study inhibited the activity of the ε-toxin as determined by one or more distinct assays. Inhibition by the three compounds appeared to be specific to ε-toxin, as none of the compounds inhibited the activity of aerolysin (data not shown). Aerolysin is another pore-forming toxin, structurally similar to ε-toxin, but does not exhibit significant amino acid sequence similarity to ε-toxin [44,45]. Informatics searches of the three compounds revealed that compound II was active in a variety of different HTS assays, including an HTS to identify inhibitors of Shiga toxin (PubChem), an HTS to identify inhibitors of Sentrin-specific proteases 6 and 8 (SENP6 and SENP8, PubChem), and an ion channel in *Plasmodium* (ChemBank) [46,47]. Compound III was active in an HTS for 14-3-3 protein interaction modulators (PubChem). No previously described activity was identified for compound I.

The activity of an optimized drug often is substantially greater than the activity of the initial hit [48,49]. It is therefore not surprising that the compounds studied were not able to provide complete protection from the cytotoxic effects of ε-toxin. However, the compounds identified in the high-throughput screen and subsequent analyses of structural analogs represent a first step at structure-activity analysis. Additional structure-activity analysis is needed to identify inhibitors with improved activity.

We hypothesize that the inhibitors interfere with the toxin pore (by inhibiting ion fluxes through the pore that otherwise contribute to cell death) or an unidentified host factor that contributes to ε-toxin-induced cytotoxicity. This hypothesis is based on our observation that none of the compounds appeared to interfere with binding of the toxin to cells or with toxin oligomerization. In contrast, compounds I and II inhibited propidium iodide influx in cells pre-treated with ε-toxin [11,12,22]. These results suggest the possibility that one or more refined structures based on the compounds identified in the present study may be effective post-exposure therapeutics. 

## Appendix

**Supplemental Table 1:** Analysis of Structural Analogs.

**Compound**	**z-score**
	34.0
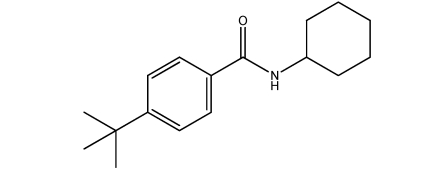
	28.1
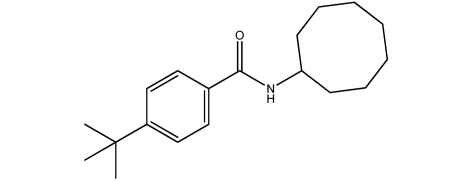
	21.3
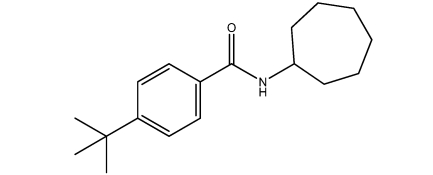
	6.6
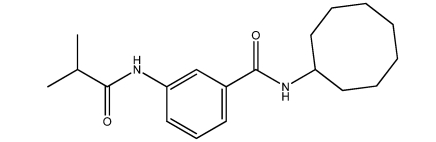
	5.1
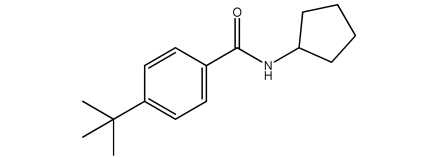
	3.8
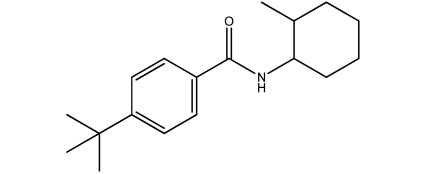
	3.8
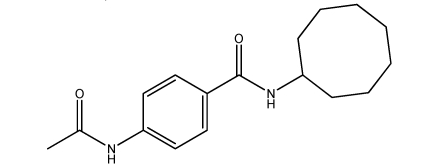
	3.6
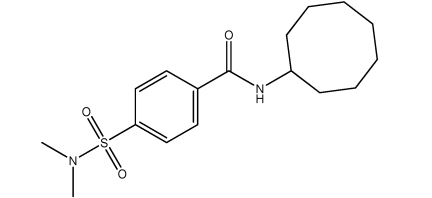
	2.1
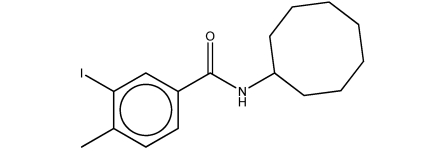
	2.1
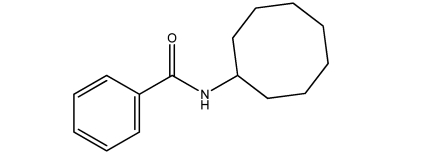
	2.0
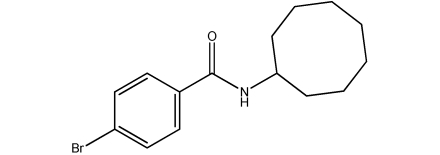
	1.5
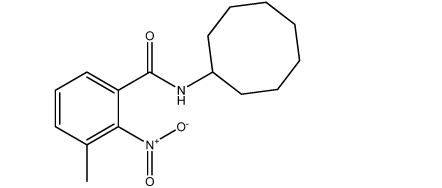
	1.3
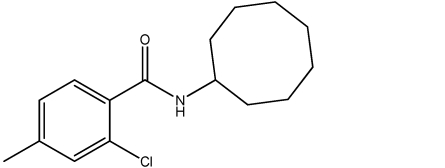
	1.3
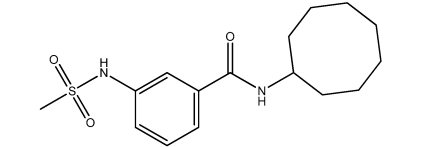
	1.1
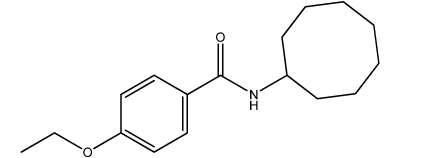
	0.9
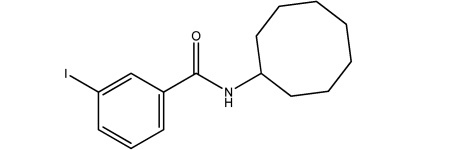
	0.6
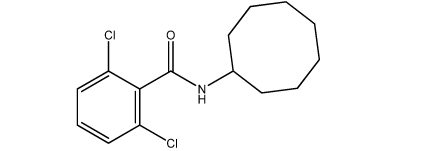
	0.5
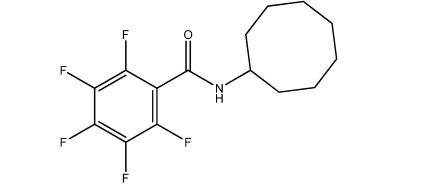
	0.4
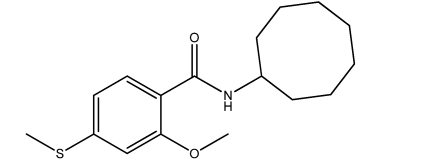
	0.3
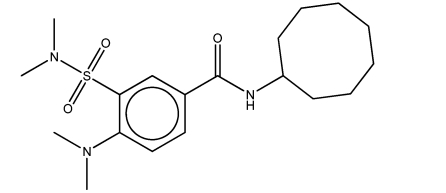
	0.2
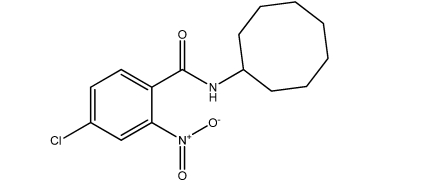
	0.2
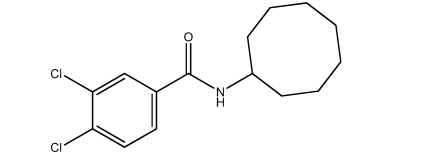
	0.1
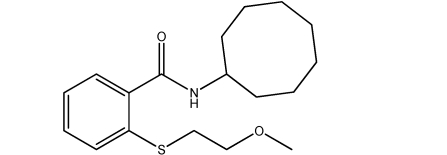
	0.0
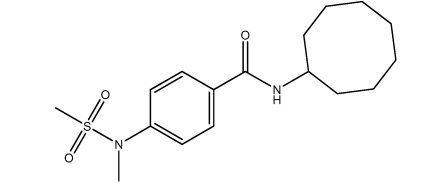
	0.0
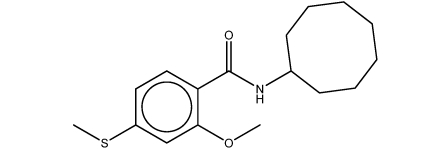
	0.0
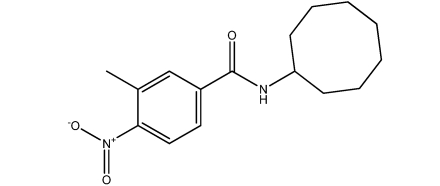
	0.0
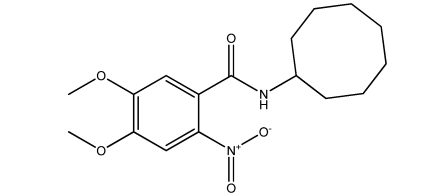
	-0.2
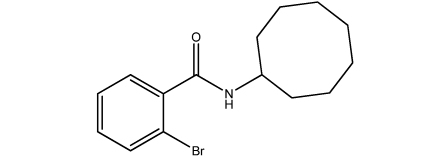
	-0.2
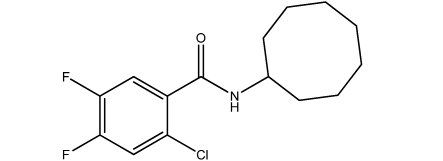
	-0.3
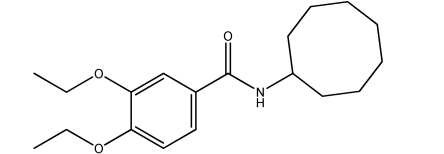
	-0.3
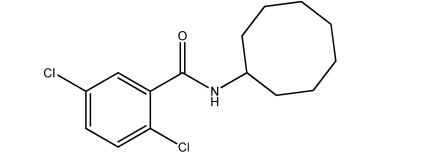
	-0.4
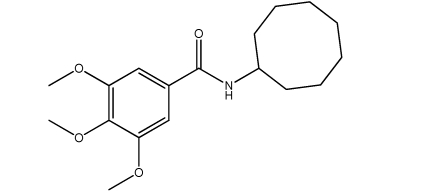
	-0.4
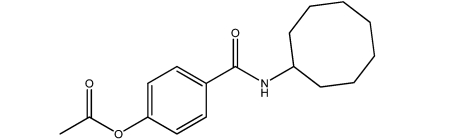
	-0.4
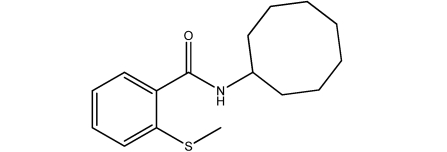
	-0.6
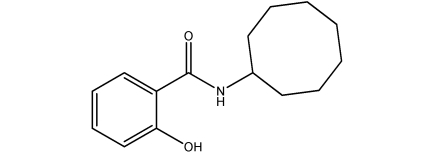
	-0.6
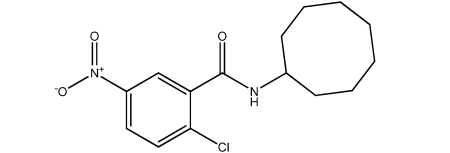
	-0.8
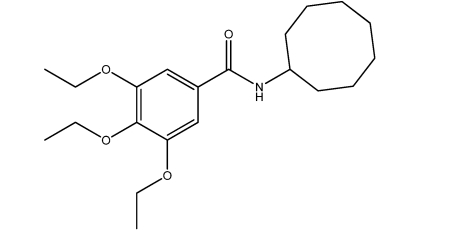
	-0.8
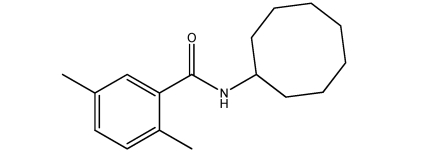
	-0.9
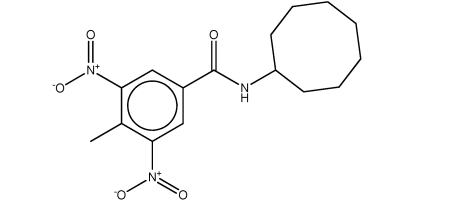
	-1.1
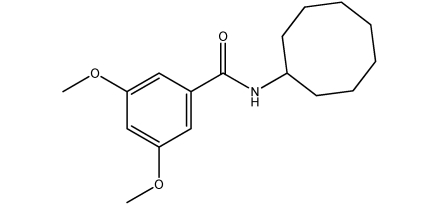
	-1.5
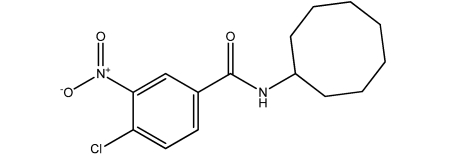
	-1.6
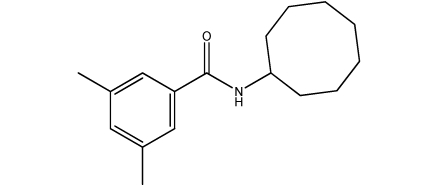
	-1.8
